# Challenges imposed by the COVID-19 pandemic on the Obstetrics and Gynecology residency program: a mixed-methods Swedish survey in the COPE Staff cohort study

**DOI:** 10.1186/s12909-022-03631-0

**Published:** 2022-08-05

**Authors:** Matilda Wådell, Anne K. Örtqvist, Karolina Linden, Magnus Akerstrom, Ola Andersson, Ylva Carlsson, Sofie Graner, Maria Jonsson, Elin Naurin, Verena Sengpiel, Malin Veje, Anna Wessberg, Mehreen Zaigham

**Affiliations:** 1 Department of Obstetrics and Gynecology, Hudiksvall Hospital, Hudiksvall, Sweden; 2 Clinical Epidemiology Unit, Department of Medicine, Karolinska Institutet, Stockholm, Sweden; 3 Department of Obstetrics and Gynecology, Visby County Hospital, Visby, Sweden; 4 Institute of Health and Care Sciences, Sahlgrenska Academy, University of Gothenburg, Gothenburg, Sweden; 5 Region Västra Götaland, The Institute of Stress Medicine, Gothenburg, Sweden; 6 School of Public Health and Community Medicine, Institute of Medicine, Sahlgrenska Academy, University of Gothenburg, Gothenburg, Sweden; 7 Pediatrics, Institution of Clinical Sciences Lund, Lund University, Lund, Sweden; 8 Department of Neonatology, Skåne University Hospital, Malmö, Sweden; 9 Region Västra Götaland, Department of Obstetrics and Gynecology, Sahlgrenska University Hospital, Gothenburg, Sweden; 10 Centre of Perinatal Medicine and Health, Institute of Clinical Sciences, Sahlgrenska Academy, University of Gothenburg, Gothenburg, Sweden; 11 BB Stockholm, Danderyds Hospital, 182 88 Danderyd, Sweden; 12 Department of Women’s and Children’s Health, Uppsala University, Uppsala, Sweden; 13 Department of Obstetrics and Gynecology, Uppsala University Hospital, Uppsala, Sweden; 14 Department of Political Science, University of Gothenburg, Gothenburg, Sweden; 15 Institute of Biomedicine, Department of Infectious Diseases, University of Gothenburg, Gothenburg, Sweden; 16 Region Västra Götaland, Department of Infectious Diseases, Sahlgrenska University Hospital, Gothenburg, Sweden; 17 Obstetrics and Gynecology, Institution of Clinical Sciences Lund, Lund University, Lund, Sweden; 18 Department of Obstetrics & Gynecology, Lund University and Skåne University Hospital, 205 01 Malmö, Sweden

**Keywords:** COVID-19, Pandemic, Education, Residency, Survey, Work environment, Obstetrics, Gynecology

## Abstract

**Background:**

To outline how the training program and work situation of residents in Obstetrics and Gynecology (OB-GYN) was affected by the pandemic and to illuminate how residents experienced these changes.

**Methods:**

As part of the COVID-19 in Pregnancy and Early Childhood Staff (COPE Staff) cohort study, between January and May 2021, all participating residents were invited to answer a 28-question online Resident Survey focusing on their specialist education, work situation and experiences during the COVID-19 pandemic. Descriptive statistics were given in percentages for categorical variables and means and standard deviations (SD) for continuous variables. Univariate comparative analyses were performed with the use of the Pearson’s Chi-2-test for dichotomous data. The association between residents’ worry about the quality and length of their specialist training, with extra clinical hours and transfer to other healthcare institutions were assessed by multivariate logistic regression. Free text responses were analyzed by content analysis.

**Results:**

Of the 162 participating OB-GYN residents, 69% expressed concern that the pandemic would have a negative impact on their training. Ninety-five (95%) reported cancellation/postponement of educational activities, 70% performed fewer surgeries and 27% had been transferred to other healthcare institutions where about half reported having gained more general knowledge as a physician. Working extra clinical hours was reported by 69% (7.4 ± 5.3 hours per week) and 14% had considered changing their profession due to the pandemic. Senior residents, compared to junior residents, more often experienced cancelled/postponed clinical rotations (30% vs 15%, *P*=0.02) and reported performing fewer surgeries (*P*=0.02). The qualitative analysis highlighted the lack of surgical procedural training as a major concern for residents.

**Conclusion:**

The COVID-19 pandemic has strongly impacted the training program and work situation of OB-GYN residents in Sweden. Residents were concerned over the negative impact of the pandemic on their training program and senior residents reported more missed educational opportunities as compared to junior residents. Program directors, head of institutions and clinical supervisors can use the problem areas pinpointed by this study to support residents and compensate for missed educational opportunities. While hands-on-training and operating time cannot be compensated for, the authors hope that the findings of the study can help develop new strategies to minimize the negative impact of the current and future pandemics on resident education and work situation.

## Highlights

• There is widespread worry among OB-GYN residents that the COVID-19 pandemic has had a negative impact on their residency program

• Significant cancellation/postponement of scheduled educational activities, reductions in surgical activities, transfer of residents to other healthcare institutions (including COVID-19 wards), reduced research time and extra hours of clinical work were reported

• Residents towards the end of their residencies were more affected as compared to junior residents regarding missed educational activities

• Strategies to minimize the negative impact of the pandemic on resident education and work situation are immediately needed

## Background

The COVID-19 pandemic has placed an enormous strain on healthcare systems, which has affected both patients and healthcare providers. For surgical residents, who are expected to achieve predetermined clinical and educational milestones, the new conditions may affect their work situation as well as their progress towards specialist competency. Studies have shown that educational activities have been cancelled due to the pandemic and that there has been a transition towards digital learning with limited clinical and surgical training [1–4]. Further, residents have been transferred to other healthcare institutions to assist in the care of COVID-19 patients [4–7].

Although there is some variation in the structure and format of residency programs throughout the world, the challenges resulting from the pandemic have been the same. In Sweden, the Obstetrics and Gynecology (OB-GYN) residency program is at least five years long and based on completing specified learning targets set by the Swedish National Board of Health and Welfare consisting of clinical practice under supervision, clinical rotations and mandatory courses [8]. This specialty combines surgical care with emergency situations, where a large portion of patient care cannot be postponed and operative procedures are a prerequisite to on-call duties. Web-based substitutes cannot fully replace clinical hands-on training [9] and residents affected by delays in their educational activities may face prolongations in their training programs.

As part of the nationwide COPE Staff cohort study [10], which has the overall purpose of investigating how the work environment for Swedish healthcare workers employed at antenatal care units, delivery departments and neonatal care units has been affected by the COVID-19 pandemic, this study focused specifically on OB-GYN residents. In collaboration with the Swedish Society for Obstetricians and Gynecologists under training (OGU) [11], the study aimed to understand the impact of the COVID-19 pandemic on the OB-GYN resident training program including surgical training and research; work situation including stress levels and comparison of effects depending on estimated year of residency completion.

## Material and methods

**Fig. 1:**
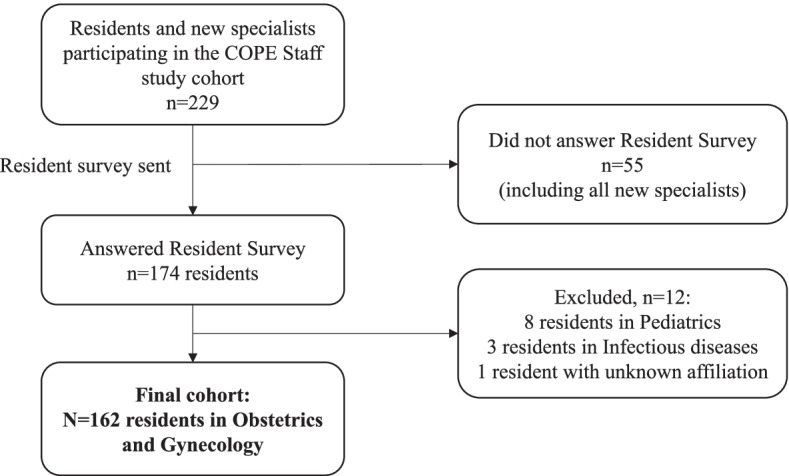
Flow chart over the study participants

The COPE Staff cohort study [10] is a cross-sectional, nationwide, online survey. Digital links to participate in the study were distributed at national OB-GYN congresses, to members of Swedish Society of Obstetrics and Gynecology (SFOG, www.sfog.se), via study program directors and through direct contact with OB-GYN residents at local hospitals. Participants who reported themselves as ‘residents’ or as ‘specialists with less than one-year work experience’, defined as ‘new specialists’, were invited to answer another survey, namely the ‘Resident Survey’ (Fig. 1).

### Survey development and distribution

The Resident Survey was designed to specifically focus on key areas of resident education including, but not limited to: surgical training, effect on medical education, research, experience of working in the pandemic and transfer to other healthcare institutions outside the home (OB-GYN) department (e.g. departments of internal medicine, infectious diseases, anesthesia and intensive care medicine, or others including specific COVID-19 wards). Based on estimated year of completion of residency, residents were categorized into ‘junior’ (planned to complete specialist training by 2023 or later) and ‘senior’ residents (planned to complete specialist training by 2021-2022). The latter group included physicians who became specialists between the first COPE Staff Survey and the Resident Survey.

The Resident Survey was developed using a two-step Delphi process [12]. A review of existing instruments led to a 28-question survey with a mixture of multiple choice and open-ended questions; thereafter a panel of OGU representatives [11] reviewed the survey in the first round of the Delphi process. During the second round, it was reviewed by the COPE Staff study steering board which consisted of a multi-disciplinary team of scientists from different fields [10].

The surveys were emailed to participants between January and March 2021. They were sent out in succession, with an approximate 4-week interval between them. This time interval was to allow respondents to not be over-burdened by surveys being sent in quick succession and to encourage completion of both surveys. Participants had the option to decline answering some demographic questions such as age, level of experience, gender, pregnancy status, or children [10]. Electronic reminders were sent three times, at one-week intervals, to those who had not responded. In addition to generic emails, individual reminders were sent to OB-GYN residents that had answered the COPE Staff Survey but not the Resident Survey one week before the closing of the Resident Survey. Once recruited, all responses were analyzed and presented in a way that individual data could not be traced back to any respondents.

### Statistical analyses

Statistical analyses were performed using SPSS (26.2) (IBM Corp, Armonk, NY). Descriptive statistics were given in counts and percentages for categorical variables and means and standard deviations (SD) for continuous variables. The association between residents’ concern about the quality and length of their specialist training to extra clinical hours and redeployment were assessed by multivariate logistic regression. The Pearson’s Chi-2-test was used to compare the effect of the pandemic on the education program and work situation of senior residents to junior residents. A two-sided *P*-value <0.05 was considered statistically significant. For comparisons between more than two groups, the Kruskal Wallis test was used.

### Qualitative analyses

The free text answers, *n*=77, were analyzed by content analysis inspired by Elo and Kyngäs [13] and presented in three sub-themes; ‘Disruptions in residency training due to the COVID-19 pandemic, ‘Support of resident training during the COVID-19 pandemic from the local healthcare institution management’ and ‘Psychological consequences for residents under training’.

## Results

### The study population

The COPE Staff study cohort included 229 residents. The Resident Survey was sent to these participants and 174 respondents answered this survey, yielding a response rate of 76%. Of these, 162 (93%) were residents in OB-GYN and were included in the final study population (Fig. 1). The background data of the study population are presented in Table 1. The total number of OB-GYN residents estimated in Sweden for the year 2020 were 456 [10], corresponding to a 36% answer frequency from the planned study population. The majority of respondents were female (75%, *n*=122), between 31-40 years of age (59%, *n*=96), brought up in Sweden (69%, *n*=111) and had children under the age of six in the household (79%, *n*=128). All six Swedish healthcare regions were represented.

**Table 1:** Background data of study participants (*N*=162)

Characteristic		***N***=162	%
Age (years)	20-30	9	6
	31-40	96	59
	41-50	12	7
	No response^a^	45	28
Healthcare region	Northern Healthcare Region	11	7
	Healthcare Region of Middle Sweden	27	17
	Stockholm – Gotland	30	19
	Southeast Healthcare Region	29	18
	Southern Healthcare Region	36	22
	West Götaland and Halland	29	18
Gender	Female	122	75
	Male	8	5
	No response^a^	32	20
Country of upbringing	Sweden	111	69
	Other Nordic Country	4	3
	Other European Country	10	6
	Non-European country	5	3
	No response^a^	32	20
Level of experience	Senior residents	56	35
	Junior residents	103	64
	No response^a^	3	1
Research experience	PhD	12	7
	Registered PhD student	9	6
	Planned PhD registering	15	9
	No research experience	122	75
Tested positive for COVID-19^b^	Yes	16	10
	No	66	41
	Not tested	80	49
Are you or your partner currently pregnant?	Yes, I am pregnant	22	14
	Yes, my partner is pregnant	2	1
	No	106	65
	No response	32	20
Participants with children within specified age groups (in years) within the household ^b^	≤6	128	79
	7-15	22	14
	≥16	1	1
	No children	41	25
	No response^a^	32	20

^a^ Missing data due to respondents not completing the COPE Staff Survey I (please see Fig. 1)

^b^ Reverse transcription quantitative polymerase chain reaction

### Effect on resident training program

Table 2 outlines the impact of the pandemic on the specialist training program as reported by the residents. Most participants stated that their education program had been affected by the COVID-19 pandemic (94%, *n*=153), with cancelled/postponed clinical rotations (OB-GYN rotations: 20%, *n*=33; Non-OB-GYN rotations: 20%, *n*=32), mandatory courses (OB-GYN courses: 38%, *n*=62; Non-OB-GYN courses: 42%, *n*=67), scheduled clinical supervision (21%, *n*=34) and other educational activities such as journal clubs/team practices/meetings/interventions (78%, *n*=127). The majority were worried that the pandemic would have a negative impact on the quality of their specialist education (69%, *n*=112), and 14% (*n*=23) had considered changing their profession due to the pandemic.

**Table 2:** Impact of the COVID-19 pandemic on the specialist training program and work situation for residents in Obstetrics and Gynecology (*N*=162)

			***N***=162	%
Will your residency be extended?	Yes		11	7
	No		120	74
	Do not know/want to answer		31	19
Effect on specialist training program^a^	OB-GYN clinical rotations were cancelled/postponed		33	20
	Non-OB-GYN clinical rotations were cancelled/postponed		32	20
	Mandatory OB-GYN courses were cancelled/postponed		62	38
	Mandatory non-OB-GYN courses were cancelled/postponed		67	42
	Planned clinical supervision was cancelled/postponed		34	21
	Other educational activities were cancelled/postponed (journal clubs, team practices, meetings, interventions)		127	78
	My education has not been affected by the pandemic		9	6
Effect on surgical training?	I have performed less surgeries than before the pandemic		113	70
	No difference		38	24
	I have performed more surgeries than before the pandemic		1	1
	Do not know/Do not want to answer/No opinion		10	6
Worked from home?	Yes		142	88
	No		20	12
Transferred to another healthcare institution, and if so, which department?	Yes^a^	Internal medicine	20	12
		Infectious diseases	13	8
		Anesthesia and intensive care medicine	11	7
		Other (COVID-19 ward, laboratory services, emergency dept)	12	7
	No		118	73
Performed clinical assignments normally performed by other professions (nurse, mid-wife or assistant nurse)?	Yes		20	12
	No		139	86
	No response		3	2
Worked at a COVID-19 ward?	Yes		49	30
	No		113	70
Worked extra clinical hours?	Yes		111	69
	No		44	27
	Do not know/do not want to answer		7	4
Effect on research activity?	I have done less research than before the pandemic		10	25
	No difference		19	48
	I have done more research than before the pandemic		6	15
	Do not know/Do not want to answer/No opinion		5	13
Support from the home healthcare institution to improve specialist training?^a^	Non-OB-GYN clinical rotations have been prioritized		82	51
	OB-GYN clinical rotations have been prioritized		73	45
	Surgical training has been prioritized		33	20
	No changes		54	33
	Other		17	11
Are you worried that the pandemic will have a negative effect on the quality of your specialist training?	Yes		112	69
	No		38	24
	Do not know/Do not want to answer/No opinion		12	7
Are you worried that your residency will be extended?	Yes		46	28
	No		104	64
	Do not know/Do not want to answer/No opinion		12	7
Have you, due to the pandemic, considered changing your profession?	No		137	85
	Yes		23	14
	Do not know/Do not want to answer/No opinion		2	1

^a^ Possible to select more than one option

More than two thirds of the respondents (70%, *n*=113), had performed fewer surgeries than before the pandemic. Of the respondents, 28% (*n*=46) reported being worried that their residency would be prolonged due to the pandemic. Eleven residents (7%) stated that they were certain that their residency would be prolonged. Of these, three were unsure of the total duration of extension whilst for the remaining eight, the expected length of the extension varied between 8 to 40 weeks (mean 18.6 ± 11.0 weeks).

The percentage of residents expressing worry for a negative effect on the quality of their specialist training were lower among residents receiving support from the home healthcare institution to improve specialist training regarding OB-GYN clinical rotations and surgical training compared to residents not receiving support (58% versus 79%, *P*=0.005, and 45% versus 75%, *P*=0.002, respectively). Support from the home healthcare institution concerning non-OB-GYN clinical rotations did not affect the level of worry for a negative effect on the quality of their specialist training (*P*=0.18). Furthermore, support from the home healthcare institution did not affect the residents’ worry about their residency being prolonged (*P*=0.57 for OB-GYN clinical rotation, *P*=0.64 for non-OB-GYN clinical rotation and *P*=0.12 for surgical training).

Research experience was reported by 25% of respondents (*n*=40). Of these, one-fourth reported a reduction in allocated research time due to the pandemic (25%, *n*=10) of which the majority were registered/soon-to-be registered PhD students or had a PhD degree (*n*=9). Most commonly, residents with research experience did not notice any changes in their research time (48%, *n*=19).

### Effect on resident work situation

**Fig. 2:**
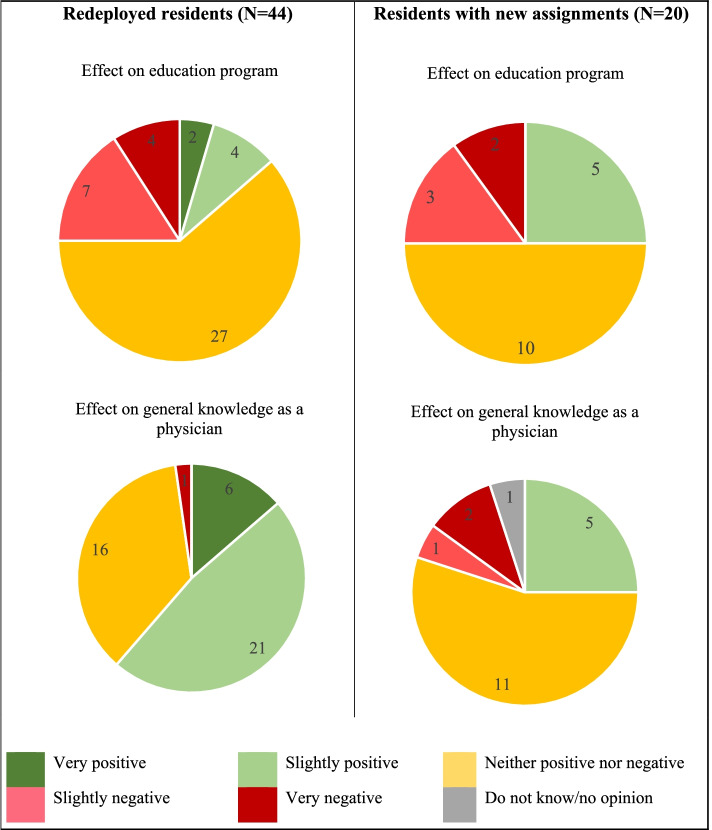
Residents-reported experience of transfer to other healthcare institutions and of new assignments normally performed by other professions

When asked if the residents had to work extra hours during the pandemic, 69% (*n*=111) of the respondents answered in the affirmative. Among those who worked extra hours, during the month with the most extra work, respondents reported on average 7.4 hours per week (SD ± 5.3 hours, range 8-40 hours per week). Of note, 30% (*n*=49) had been transferred to work in a COVID-19 ward, with the length of redeployment varying widely from less than one week to 27 weeks. Almost half of the residents (23/49, 47%) were transferred for 1-4 weeks (Table 3). As a separate question, the respondents were also asked how they had experienced being transferred to another healthcare institution. Forty-four (*n*=44) answered in relation to overall reported ‘stress level’. ‘Very increased’ stress levels were reported by 5% (*n*=2), 39% (*n*=17) reported ‘slightly increased’, 50% (*n*=22) ‘neither increased nor decreased’ and 7% (*n*=3) ‘slightly decreased’. With regards to the effect of healthcare institution transfer on their medical education program and on their general knowledge as a doctor, most reported that it neither had positive nor negative effects on their educational program but a slightly positive effect on their general knowledge as a doctor (Fig. 2).

**Table 3:** Multivariate logistic regression analysis for the association between residents’ worry for a negative effect on the quality of their specialist training or worry for their residency being prolonged and working extra clinical hours, being transferred to another healthcare institution, and working on a COVID-19 ward

		Worry for negative effect on the quality of their specialist training			Worry for their residency being prolonged		
Covariates	n (%)	P (%)	b	*P*-value	P (%)	b	*P*-value
Model 1: Worry for negative effects = constant + working extra clinical hours + working on a COVID-19 ward							
Constant			0.64	0.06		-1.6	0.2
Working extra clinical hours							
Yes	111 (72)	74	0.64	0.1	21	0.58	0.1
No	44 (28)	59	ref		32	ref	
Being redeployed to another healthcare institution working on a covid-19 ward							
Yes, > 8 weeks	4 (3)	75	-0.001	1.0	25	-1.4	0.4
Yes, 5-8 weeks	12 (7)	67	0.11	0.9	8	0.88	0.5
Yes, 1-4 weeks	23 (14)	70	-0.30	0.6	39	0.17	0.9
Yes, <1 week	10 (6)	70	0.77	0.5	30	0.29	0.8
No	113 (70)	69	ref		28	ref	
Model 2: Worry for negative effects = constant + working extra clinical hours + being transferred to another healthcare institution							
Constant			0.72	0.04		-1.3	<0.001
Working extra clinical hours							
Yes	111 (72)	74	0.75	0.07	21	0.65	0.1
No	44 (28)	59	ref		32	ref	
Being transferred to another health care institution							
Yes	44 (27)	69	-0.57	0.2	30	0.03	0.9
No	113 (73)	71	ref		28	ref	

*n* number of respondents in the total study population, *P* Prevalence (%) reporting worry for decreased quality of residency and prolonged residency, respectively, b=odds ratio expressing worry for decreased quality of residency and prolonged residency versus not expressing worry

During the COVID-19 pandemic, 12% (*n*=20) of the respondents had performed clinical assignments usually performed by other professions. The respondents reported that their newly allocated assignments usually were performed by nurses (*n*=3), assistant nurses (*n*=17) or midwives (*n*=3). When asked how these new assignments affected the respondents’ stress level compared to before, 15% (*n*=3) reported ‘very increased’ stress levels, 15% (*n*=3) ‘slightly increased’ and the remaining 70% (*n*=14) neither increased nor decreased. Most reported that the new assignments had neither positive nor negative effect on their education program and their general knowledge respectively (Fig. 2).

Table 3 explores the association between residents’ worry for a negative effect on the quality of their specialist training or worry for their residency being prolonged to working extra clinical hours, being transferred to another healthcare institution outside OB-GYN, or working at a COVID-19 ward. No significant associations were found. Of the residents who had worked extra clinical hours during the pandemic (72%, *n*=111), 74% were worried that the pandemic would have a negative effect on the quality of their specialist education, compared to 59% of those not working extra clinical hours (28%, *n*=44) (*P*=0.1, when included in multivariate regression models together with being transferred to another healthcare institution or working at a COVID-19 ward). Among respondents who had worked extra clinical hours during the pandemic, 21% were worried that their residency would be prolonged, compared to 32% among those who had not worked extra hours (*P*=0.1). Being transferred to another healthcare institution or working at a COVID-19 ward did not affect the residents’ worry for a negative effect on the quality of their specialist training or worry for their residency being prolonged (Table 3).

### Comparison between senior and junior residents

Comparisons between senior and junior residents are shown in Table 4. Senior residents had more cancelled/postponed OB-GYN clinical rotations (30% versus 15%, *P*=0.02), non-OB-GYN clinical rotations (39% versus 10%, *P*<0.01) and mandatory courses specific for OB-GYN (48% versus 32%, *P*=0.04) as compared to junior residents. They also reported performing fewer surgeries (80% versus 64%, *P*=0.02) as compared to junior residents. With regards to prolongation of residency due to the pandemic, there were no significant differences between the groups (*P*=0.12).

**Table 4:** Comparison of the effect of the COVID-19 pandemic on the specialist training program and work situation of senior vs junior residents

			Senior residents ***N***=56 (%)	Junior residents ***N***=103 (%)	***P***-value Pearson Chi-2-test
Will your residency be extended?		Yes	6 (13)	4 (5)	0.12
		No	42 (88)	77 (96)	
Are you worried that the pandemic will have a negative effect on the quality of your specialist training?		Yes	42 (79)	67 (71)	0.29
		No	11 (21)	27 (29)	
Are you worried that your residency will be extended?		Yes	15 (29)	28 (30)	0.94
		No	37 (71)	67 (71)	
Have you, due to the pandemic, considered changing your profession?		Yes	10 (18)	12 (12)	0.30
		No	46 (82)	89 (88)	
How was your training affected by the pandemic?	OB-GYN clinical rotations were cancelled/postponed	Yes	17 (30)	15 (15)	0.02
		No	39 (70)	86 (85)	
	Non-OB-GYN clinical rotations were cancelled/postponed	Yes	22 (39)	10 (10)	<0.01
		No	34 (61)	91 (90)	
	Mandatory OB-GYN courses were cancelled/postponed	Yes	27 (48)	32 (32)	0.04
		No	29 (52)	69 (68)	
	Mandatory non-OB-GYN courses were cancelled/postponed	Yes	22 (39)	42 (42)	0.78
		No	34 (61)	59 (58)	
	Other educational activities cancelled /postponed (Team trainings, intervention trainings, meetings, journal clubs)	Yes	44 (79)	79 (78)	0.96
		No	12 (21)	22 (22)	
	Planned clinical supervision was cancelled/postponed	Yes	13 (23)	18 (18)	0.41
		No	43 (77)	83 (82)	
Have you performed fewer surgeries due to the pandemic?		Yes	45 (80)	66 (64)	0.02
		No/Do not know	11 (20)	37 (36)	
Have you been transferred to another healthcare institution?		Yes	11 (20)	31 (31)	0.15
		No	43 (80)	68 (69)	
Have you been required to work extra at your healthcare institution due to the pandemic?		Yes	42 (78)	67 (68)	0.19
		No	12 (22)	32 (32)	

#### Qualitative analysis

##### Disruptions in residency training due to the COVID-19 pandemic

Qualitative analysis of the 77 free text answers revealed a challenging situation for the residents. The COVID-19 pandemic had direct consequences on their resident training. Due to the restrictions, residents had to stay home if they had any symptoms which resulted in multiple sick days and days off work due to taking care of their sick children. One participant described their situation: *“I work at a small hospital where we were already short staffed [before the COVID-19 pandemic]. Having colleagues call in sick or staying home to look after their sick children has affected my work a lot and has kept me from progressing in my residency training”.* Planned courses were initially cancelled all together, and later on in the pandemic, replaced with digital versions. One participant reported that their residency would be prolonged by several months due to cancelled courses and a lack of essential course certificates. Surgical rotations were abruptly cancelled for several participants since all elective surgery, which included benign conditions, were stopped in the beginning of the pandemic to redirect healthcare to COVID-19 patients. For example, one participant wrote: *“Out of a two-month long surgical rotation, I only got to spend five days in the operating theater and all cases were super complicated”.* Further, one OB-GYN healthcare institution excluded all residents from performing surgeries since they took longer time and occupied the operation staff for a longer period.

Due to the risk of gathering groups of people, participants were excluded from meetings and gatherings that they normally would have attended. For example, internal meetings and training sessions were cancelled or held in smaller groups due to the risk of infection. One participant wrote: *‘Almost all meetings were cancelled, even case descriptions that we really needed to take part of”.* In addition, scientific report writing, which is an essential part of residency training, was also delayed due to the supervisors not having enough time for research. The participants also missed opportunities for networking with peers and potential future employers. Meeting patients in digital meetings or over the phone affected their consultation skills negatively.

##### Support of resident training during the COVID-19 pandemic from the local OB-GYN healthcare institution management

Some participants described that there was a lack of support during their resident training, others acknowledged that they received some support from their local OB-GYN healthcare institution management despite the challenging situation of the pandemic. Such initiatives included structured follow-up and progress reports with plans on how to mediate difficulties that arose due to the pandemic and provision of local residency training via digital platforms. Further, some OB-GYN healthcare institutions tried to relocate residents internally rather than send them to other departments in the hospital if there were staff shortages. For example, one participant shared: *“[I have received] less time for planned rotations, but they have tried to let me go when the situation allowed for it”.* Another participant described that their healthcare institution tried to carry on with their ordinary residency training program but that they lacked any plan for how the residents could make up for missed training, leaving them without enough support. One resident wrote that the local residency director tried to make sure that the residents took part in the few surgeries that occurred: *“Everyone has supported us so that we can assist or perform the procedures that take place”.*

##### Psychological consequences for residents under training

Since there were staff shortages and a great influx of COVID-19 patients that needed hospitalization, there was a net shortage of available hospital beds. This caused ethical concerns for the participants, and they worried about patients not receiving optimal care. Some participants received psychological care from psychologists due to stressful and traumatic COVID-related incidents in the workplace. A shortage of healthcare workers/colleagues and the constantly changing work schedule affected the family life of participants to the point where some considered changing their specialty to one that is more adaptable to family life. Participants ventilated concerns that they were used solely on the basis to keep up with the running demands of the department rather than as future specialists and consultants. This caused major worry and concern among residents since they felt that they may not be well enough trained in the future to provide good care to their patients. One participant stated: *“My home department has recommended that I should be promoted to a consultant but I don’t think I meet the requirements due to my lack of surgical training”.* A lack of available consultants to support residents resulted in participants sometimes filling positions in the department that they did not feel qualified to handle. One participant expressed that *“you feel lucky if there is a consultant [available] to ask for advice [during a shift]”.*

Some participants were transferred to COVID-19 wards and this disrupted their residency program. This had emotional consequences as several participants worried about how it would affect their residency training. For others, the sudden disruption triggered concerns regarding whether they had chosen the right specialty altogether, since they were given the opportunity to try another specialty which they may prefer over OB-GYN. One resident wrote: *“It has been positive for me to transfer to the department of internal medicine, I have been inspired, and am now questioning my choice of speciality”.*

## Discussion

### Main findings

This nationwide, cross-sectional, mixed-methods survey studied the effect of the COVID-19 pandemic on the OB-GYN resident training program in Sweden. We identified several key areas of concern including an overall perceived reduction in educational activities, transfer to other healthcare institutions outside the field of OB-GYN and wide-spread worry among residents that the pandemic would have a negative impact on their training program. We found that the educational and surgical activities of senior residents were more affected as compared to junior residents. One-in-seven residents had considered changing their profession due to the pandemic which is an important issue that needs to be addressed in future studies.

### Implications

To the best of our knowledge, this is the first study describing the Swedish resident training program and work situation during the COVID-19 pandemic as well as the first mixed-methods study on the topic. Comparable effects have been confirmed by several international studies. Reporting the opinion of almost 500 OB-GYN residents from Italy, Bitonti et al. [3] found that more than half of the respondents had observed a significant decrease in their educational activities. Similarly, in a survey sent to surgical residents and early-career surgeons in the United States, 96% responded that the pandemic was having a negative impact on their clinical experience [2]. Both the qualitative and the quantitative analyses in our study revealed widespread reductions in surgical activity, which aligns well with previous studies [1, 2, 4, 5, 7, 14, 15]. However, these findings are not surprising considering reports from Swedish OB-GYN healthcare registers estimating an approximate 30% reduction in benign surgical cases in 2020 as compared to the previous year and the study was carried out in the middle of a wave of COVID-19 cases in Sweden [16]. Nevertheless, these findings need to be considered when planning pragmatic steps to mitigate the lack of surgical training opportunities for residents in the current and future pandemics. For example, options for remote surgical training could be considered as described by Hoopes et al. [17], including laparoscopic box trainers, online surgical simulation modules or other e-learning tutorials and videos [18]. In addition, support from the home institution was associated with a lower level of worry for negative effects on the quality of the specialist training. Extra support from the home institution may therefore be an important way to reduce the impact of the COVID-19 pandemic on the OB-GYN residency training program or in other situations where the healthcare organizations are under pressure.

In studies from the United States [2], Brazil [5] and Europe [4], reports of redeployment of residents to other healthcare institutions, including designated COVID-19 wards, have varied between 15-35%, which aligns well with the proportion in our study (30%). Being removed from one’s field of specialization and working in another environment can be stressful, which was echoed by some of the participants in this study describing concern over how redeployment would affect their residency training. The pandemic has affected mental well-being and increased levels of burnout among healthcare workers [19, 20], but notably, almost half of the transferred residents in our study had experienced a positive effect on their general knowledge as a physician. A similar effect was seen by Boekhurst et al. [4] where transferred residents reported having gained further skills in Internal and Critical care medicine. A recent systematic review [21] cemented the findings of our study by concluding that the pandemic had had a profound effect on residency programs globally, particularly in surgical and interventional medical fields.

Psychological consequences were described by the residents in our study, including negative impact on family life, ethical stress due to suboptimal care of the patients and a lack of support from consultants. A meta-analysis regarding psychological effects on healthcare personnel during viral outbreaks showed that lack of practical support was a contributing risk factor for psychological distress [22]. Other risk factors included being younger in age, being more junior and being the parents of dependent children [22]. As evident in the demographics of our study population, a large number of our participants fall into these risk factors. Further investigation regarding burnout and stress among residents would therefore be an important future area of research.

Our study explored changes in research activity for residents with prior research experience. The affected residents were predominantly PhD students (planned or registered) and one fourth reported an overall reduction in scheduled research time due to the pandemic. Since research time is often financed by separate grants, these changes can possibly lead to the non-use of important research funding and delay planned completion of PhD studies [23, 24].

We focused on comparisons between senior and junior residents since senior residents have a shorter time left in achieving specialist competence, and therefore have smaller margins for eventual changes and delays. Our findings showed that senior residents were significantly more likely to have experienced clinical rotations being cancelled or postponed. One explanation for this finding could be that many clinical rotations within resident training programs are planned towards the end of the residency. During the pandemic, senior residents were therefore more likely to have their rotations cancelled/postponed as compared to junior residents. Senior residents may have been considered “more valuable” for their departments because of their added clinical experience as compared to junior residents. Healthcare institution management may therefore have been more likely to retain them as compared to junior residents to help keep up the running of the department. An important implication of these findings is the resulting delay in senior residents from achieving their specialist degrees on time, thereby extending their residencies. It is therefore vital that program directors employ measures to prevent future shortages of specialists within OB-GYN and other specialties.

### Strengths and limitations

The cross-sectional format, electronic distribution and comprehensive design of the COPE Staff study were some of the major strengths of the current study. A high response rate to the Resident Survey and inclusion of residents from all geographical regions in Sweden were additional strengths. However, since it was possible to submit the survey without completing all questions, there was some missing data which could not be accounted for and this affected the power of the analyses. Despite a high response rate, our study was able to sample approximately 36% of the target population resulting in a risk of selection bias. However, whether those who experienced effects of the pandemic were more likely to respond to the survey, or the opposite, remains unclear and this number includes residents on parental/sick leave and the total number of residents can therefore be less than reported. Nevertheless, we obtained the views of 36% of the total population which is a percentage higher than those reported by similar studies [1, 5].

## Conclusion

Our study showed that the COVID-19 pandemic has strongly impacted the training program and work situation for OB-GYN residents in Sweden. We found wide-spread worry among residents and, in general, senior residents were more affected as compared to junior residents regarding missed educational opportunities and clinical rotations. Support from the home institution regarding OB-GYN clinical rotation and surgical training during the pandemic was associated with decreased worry for the resident´s specialist training. Program directors and clinical supervisors can use the problem areas pinpointed by this study to support residents and compensate for missed educational opportunities. While hands-on-training and operating time cannot be compensated for, the authors hope that the findings of the study can help find new strategies to minimize the negative impact of the COVID-19 pandemic and future pandemics on the resident training program and work situation.

## Data Availability

The datasets generated and/or analysed during the current study are not publicly available due participant informed consent formulated during the design of the study. Requests can be made to the Principle Investigator of the COPE Staff study (KL) via the corresponding author and will be discussed on a case-to-case basis.

## Abbreviations

COPE Staff: COVID-19 in Pregnancy and Early childhood Staff
COVID-19: Coronavirus disease 2019
OB-GYN: Obstetrics and Gynecology
